# Placenta accreta spectrum disorders—experience of management in a German tertiary perinatal centre

**DOI:** 10.1007/s00404-020-05875-x

**Published:** 2020-12-07

**Authors:** Anja Bluth, Axel Schindelhauer, Katharina Nitzsche, Pauline Wimberger, Cahit Birdir

**Affiliations:** Department of Gynecology and Obstetrics, University Hospital Carl Gustav Carus, Technische Universität Dresden, Fetscherstraße 74, 01307 Dresden, Germany

**Keywords:** Placenta accreta spectrum, Peripartum haemorrhage, Caesarean hysterectomy, Maternal morbidity

## Abstract

**Purpose:**

Placenta accreta spectrum (PAS) disorders can cause major intrapartum haemorrhage. The optimal management approach is not yet defined. We analysed available cases from a tertiary perinatal centre to compare the outcome of different individual management strategies.

**Methods:**

A monocentric retrospective analysis was performed in patients with clinically confirmed diagnosis of PAS between 07/2012 and 12/2019. Electronic patient and ultrasound databases were examined for perinatal findings, peripartum morbidity including blood loss and management approaches such as (1) vaginal delivery and curettage, (2) caesarean section with placental removal versus left in situ and (3) planned, immediate or delayed hysterectomy.

**Results:**

46 cases were identified with an incidence of 2.49 per 1000 births. Median diagnosis of placenta accreta (56%), increta (39%) or percreta (4%) was made in 35 weeks of gestation. Prenatal detection rate was 33% for all cases and 78% for placenta increta. 33% showed an association with placenta praevia, 41% with previous caesarean section and 52% with previous curettage. Caesarean section rate was 65% and hysterectomy rate 39%. In 9% of the cases, the placenta primarily remained in situ. 54% of patients required blood transfusion. Blood loss did not differ between cases with versus without prenatal diagnosis (*p* = 0.327). In known cases, an attempt to remove the placenta did not show impact on blood loss (*p* = 0.417).

**Conclusion:**

PAS should be managed in an optimal setting and with a well-coordinated team. Experience with different approaches should be proven in prospective multicentre studies to prepare recommendations for expected and unexpected need for management.

## Introduction

Abnormally adherent and invasive placenta, defined as placenta accrete spectrum (PAS) disorders, can complicate deliveries by unexpected massive bleeding [1]. With an incidence of about 0.17%, few obstetricians achieve routine in the management of PAS [2]. In this entity, the attempt to remove the placenta can lead to tearing of large vessels due to the lack of a detachment layer to the myometrium [3]. It is assumed that a decidua defect creates an invasive niche, particularly in the area of uterine scars [4]. The incidence of PAS has been increasing for decades [5].

The risk of PAS disorders correlates with the number of previous caesarean sections (CSs) [6, 7]. The detection of further significant risk factors such as placenta praevia has made targeted screening possible [8, 9]. Sonographic markers for PAS disorders were found and standardised [10]. Prenatal diagnosis has been shown to reduce the associated morbidity, particularly caused by peripartal haemorrhage [11]. However, the prepartum diagnosis of placenta accreta in ultrasound is still limited compared to placenta increta or percreta [12], and in individual cases, devastating bleeding occurs when a regular attempt is made to remove unknown PAS in the absence of risk factors [13]. Optimal conditions for delivery in PAS have been described, including thorough prenatal consultation and management by experienced multidisciplinary teams in large perinatal centres [14, 15]. According to current knowledge, the expertise of a well-coordinated team seems to be more decisive than the specific type of procedure [16, 17].

While there is agreement on the optimal setting for therapy in PAS, the preferred approach currently remains the result of individual experience. Not infrequently, emergency solutions have been translated into innovative, further pursued treatment methods [18–21]. In addition to the often used primary caesarean section hysterectomy, the guidelines mention several initial or definitive uterine-preserving treatment options, including leaving the placenta in situ after child development with delayed placental removal or expectant management [22, 23]. There is still no standard that contains detailed recommendations for the preferred management in various clinical situations [24, 25]. In order to prepare prospective clinical trials in this respect, a detailed description of the status quo of the PAS problem and the strategies currently applied in experienced treatment centres to solve is essential.

Therefore, we analysed cases of PAS disorders treated in a tertiary perinatal centre over a period of 7 years, focusing on risk factors, diagnosis and clinical treatment outcomes. Different individual management approaches were compared regarding blood loss and hysterectomy rate.

## Materials and methods

A monocentric retrospective cohort analysis was performed. From the total number of births at the University Hospital in Dresden, Technische Universität Dresden, Germany, between July 2012 and December 2019, all pregnancies complicated by PAS disorders that led to deliveries were identified. The cases were collected via a targeted search based on the coding of the International Statistical Classification of Diseases and Related Health Problems (ICD-10). The search terms included ‘placenta accreta’, ‘placenta increta’ and ‘placenta percreta’. Patient records, surgical records, an electronic patient database (Orbis^®^ Database, Agfa HealthCare, Bonn, Germany) and an ultrasound database (ViewPoint^®^, General Electronic Company, Boston, MA, USA) were used to confirm the diagnosis and to collect data. Patient data were anonymised and checked for possible invalid data before data analysis was started.

Cases of retained placenta were excluded, defined as a placenta that was trapped in the uterus after spontaneous separation due to constriction of the cervix. Histological confirmation was not a mandatory inclusion criterion.

Based on the literature, our data were collected on patients’ demographics, maternal medical history, obstetric and gynaecological history, prenatal findings and comorbidities, time and place of diagnosis, time and indication for delivery, management plan and surgical procedures performed, details of the operative approach (e.g. types of incisions, type of hysterectomy, duration of surgery from incision to closure), surgical findings, estimated intraoperative blood loss, additional treatment procedures (e. g. preoperative placement of ureteral stents, admission to intensive care, transfusion of blood products intraoperatively and within 48 h postoperatively, administration of tranexamic acid, antibiotics), postpartum care, length of hospital stay, necessity of delayed curettage and neonatal outcomes (e. g. birth weight, APGAR score, umbilical cord artery pH, admission to intensive care and length of stay).

PAS disorders were retrospectively staged by clinical or surgical findings at delivery according to the published clinical classification for PAS disorders by the International Federation of Gynecology and Obstetrics (FIGO) in 2019 [26]. For a case to be included as placenta accreta, it had to be mentioned in the surgical report: “no separation with oxytocin and controlled cord traction, heavy bleeding from the placental implantation site after attempts at manual removal or requiring surgical procedures”. In case “abnormal macroscopic findings over the placental bed and significant hypervascularity” were described, we evaluated the case as placenta increta. In case “placental tissue seen to be invading through the uterine serosa or into other organs” was documented, we stated placenta percreta. The surgeon's assessment had to include these criteria, albeit not literally, but rather in essence.

Ultrasound scans were analysed regarding time of diagnosis. Prenatal in-house diagnosis was considered if sonographic findings such as myometrial thinning, abnormal placental lacunae, placental bulge, hypervascularity or bridging vessels were described. External diagnosis was considered in cases referred to the study site for further diagnostics or treatment in connection with an assumed PAS disorder. In cases where PAS was not suspected prior to delivery, the diagnosis was made intrapartum.

The intended and actual type of management was analysed. If PAS was suspected, the usual protocol included multidisciplinary consultation with an experienced obstetrician, an experienced surgical gynaecologist, an anaesthesiologist, a neonatologist and a specialist in transfusion medicine. The patient was counselled extensively to agree a consensual management plan that met the patient’s individual needs and wishes, such as whether or not the uterus should be preserved. Options included: (1) CS with planned hysterectomy, (2) attempting placental removal with immediate hysterectomy in case of bleeding and (3) leaving the placenta in situ with delayed hysterectomy or (4) uterus-preserving management. The actual type of management was recorded as (1) vaginal delivery and curettage, (2) CS with placental removal with/without curettage, (3) CS with leaving the placenta in situ with or without delayed curettage or hysterectomy, (4) CS with planned hysterectomy and (5) CS with attempted placental removal with immediate hysterectomy.

Attempted or omitted placental separation was analysed. The attempt to remove the placenta was defined as a manual or operative procedure that aims to separate placental tissue from the uterus beyond simple umbilical cord traction.

The observed intraoperative blood loss was estimated taking into account the suction volume, surgical pads and swabs. In cases of acute bleeding during puerperium, the recorded blood loss during further surgical procedures was added to the previous blood loss. During the study period, several features of patient blood management were institutionally implemented. Preoperatively, oral iron was usually substituted below a haemoglobin level of 7 mmol/l. In case of suspected PAS, prepartal blood transfusion was considered at a preoperative haemoglobin level below 5 mmol/l, and at least two to four units of packed red blood cell concentrates (each approximately 300 ml) were prepared to be immediately available during surgery. The overall transfusion approach followed the interdisciplinary guideline for the use of blood products of the German Medical Association [27], according to which a blood transfusion is primarily triggered by the impaired condition of the anaemic patient or by restricted tissue oxygenation. Therefore, above an absolute transfusion threshold of 3.7 mmol/l of haemoglobin, each transfusion is largely a result of an individual evaluation of the clinical condition. Haemostatic management, including early application of tranexamic acid, as well as substitution of single haemostatic factors based on rotational thromboelastometry (ROTEM^®^), was initially performed as indicated by the treating anaesthetists and recently according to current guidelines [28]. Uterotonics were used in all patients after delivery of the foetus. Initially, oxytocin (5 IU) was applied and repeated in case of further bleeding (5–10 IU). If blood loss increased to more than > 1000 ml, oxytocin was substituted by sulprostone infusion according to uterine contraction response. Approaches of interventional radiology, such as temporary iliac/uterine arterial occlusion, were not considered in our centre due to logistical reasons.

Postpartal anaemia was graded according to the Common Terminology Criteria for Adverse Events [29].

The primary outcome was the maternal morbidity due to hysterectomy. Secondary endpoints included risk factors, prenatal detection rate and intraoperative blood loss.

The study was approved by the Research Ethics Commission of the Faculty of Medicine, Technische Universität Dresden, Germany.

Descriptive statistics were used to describe the cohort. A nonparametric test (Mann–Whitney U-test) was used for comparisons between sub-groups. Probability values were considered significant if they were less than 0.05. All statistical analyses were performed with SPSS (IBM SPSS Statistics 25, Armonk, NY, USA).

## Results

Between July 2012 and December 2019, the total number of births at the University Hospital Dresden was 18,476. As a result of the targeted case search, 56 patients with a diagnosis of PAS were documented. After detailed analysis, 4 cases reporting only placental retention were excluded. 4 other cases were excluded due to miscarriage before reaching viability. According to the surgical report, two patients had increased bleeding because of uterine atonia rather than PAS and were excluded. The inclusion criteria were met by 46 patients, resulting in an incidence of 2.49 PAS disorders per 1000 births at the study centre.

Placenta accreta was diagnosed in 26 patients (56%), placenta increta in 18 cases (39%) and placenta percreta in 2 deliveries (4%). The characteristics of the cohort studied are summarised in Table 1. In 54% (*n* = 25) of the cases, histopathological findings were available, and in 17 cases, PAS was confirmed histopathologically. In 44% (*n* = 11) of the cases analysed, the degree of PAS was consistent with the clinical diagnosis. The median (25th percentile, 75th percentile) gestational age at delivery was 37 (34, 39) weeks. 45 patients had singleton pregnancies; one patient had a twin pregnancy. In one third (33%) of the patients, an association with placenta praevia and in 19 (41%) cases a history of CS was found. One quarter (24%) of the women had a history of CS and a current placenta praevia. More than half of the women (52%) had at least one previous curettage.

**Table 1:** Cohort data: demographic and obstetric characteristics of women with PAS

	Women with PAS (*n* = 46)
Maternal age [years], median (Q1, Q3)	31 (27, 35)
Gravity, median (Q1, Q3)	3 (1, 4)
Parity (before delivery), median (Q1, Q3)	1 (0, 2)
Gestational age at delivery [weeks], median (Q1, Q3)	37 (34, 39)
Previous caesarean section, *n* (%)	19 (41)
0	27 (59)
1	10 (22)
2	5 (11)
3	1 (2)
4	3 (7)
Placenta praevia, *n* (%)	15 (33)
History of CS and present placenta praevia, *n* (%)	11 (24)
Previous curettage, *n* (%)	24 (52)
0	22 (48)
1	13 (28)
2	7 (15)
3	4 (9)
History of myoma enucleation, *n* (%)	3 (7)
Placental insufficiency, *n* (%)	5 (11)
Histopathological analysis, *n* (%)	25 (54)
PAS confirmed, *n* (% of analysed cases)	17 (68)
Grading of PAS confirmed, *n* (% of analysed cases)	11 (44)

*PAS* placenta accreta spectrum, *CS* caesarean section, *Q1* 25th percentile, *Q3* 75th percentile

The prenatal detection rate was 33% (*n* = 15) for all cases of PAS and 78% (*n* = 14) for placenta increta. One of the two women with placenta percreta had suspected PAS, and all cases of placenta accreta were unknown. When PAS was diagnosed prenatally, this was at a median of 23 (20, 30) weeks of pregnancy. The majority of diagnosis (*n* = 42; 91%)—prenatal and intrapartum—were made in our department. 4 cases were prenatally referred with suspicion of PAS between 26 and 33 weeks of gestation.

The median prenatal length of stay after admission to hospital was one (1, 20) day. Twelve women (26%) had an emergency delivery. The indications were antepartum haemorrhage (*n* = 3), threatened preterm labour (*n* = 2), premature rupture of the membranes (*n* = 2), suspected uterus rupture (*n* = 2), suspected foetal distress (*n* = 1), transverse position (*n* = 1) and birth arrest (*n* = 1).

The different modes of delivery and surgical procedures for management of PAS are shown in Table 2. In all patients with vaginal delivery, PAS was unknown prenatally and was only diagnosed postpartum during curettage. In no patient with suspected PASD, a vaginal delivery was planned or performed. The CS rate was 65% (*n* = 30) and hysterectomy rate 39% (*n* = 18). All hysterectomies performed were total hysterectomies. In more than half of the women (*n* = 8) with prenatally diagnosed PAS disorders, ureteral stents were inserted preoperatively as a prophylactic measure. Eight (17%) women had elective or emergency CS with placental removal and uterus preservation. One of them needed curettage in the puerperium. In three cases with CS with remaining placenta in situ, postpartum curettage was necessary, which was performed after a median of 19 (18, 67) days. In four cases with CS with remaining placenta in situ, the uterus could be preserved. In one case, a delayed hysterectomy 8 weeks after delivery was necessary due to extensive bleeding. In seven (15%) other patients, emergency hysterectomy was performed immediately or within 24 h after delivery by CS due to bleeding. There was no significant difference in the rate of hysterectomy when comparing attempted or deferred placental removal in known or unknown cases of PAS (*p* = 0.169).

**Table 2:** Management of women with PAS: delivery approach, details of surgical procedures

Delivery mode, (*n* = 46)	Women with PAS, *n* (%)
Vaginal delivery and curettage	16 (35)
CS with placental removal	8 (17)
Postpartum curettage when placenta removed	1 (2)
CS with placenta left in situ	5 (11)
Postpartum curettage when placenta left in situ	3 (7)
CS with planned hysterectomy	10 (22)
CS with immediate hysterectomy	7 (15)
CS with delayed hysterectomy	1 (2)
Total attempted placental removal	32 (70)
Duration of surgery	
< 1 h	23 (50)
1–2 h	13 (28)
> 2 h	10 (9)

Details of surgical procedures, (*n* = 30)	Women with PAS and laparatomy, *n* (%)
Preoperative insertion of ureteral stents	8 (27)
Skin incision	
Midline	12 (40)
Transverse	18 (60)
Uterine incision	
Fundus	11(37)
Vertical	5 (17)
Transverse	14 (47)

*PAS* placenta accreta spectrum, *CS* caesarean section, *Q1* 25th percentile, *Q3* 75th percentile

Maternal intrapartum and postpartum morbidity and neonatal outcomes are presented in Table 3. For all patients, the median-estimated intraoperative blood loss was 1600 (1100, 2750) ml. Blood transfusion was given intraoperatively and within 48 h postoperatively in 25 (54%) patients. A median number of 2 (0, 4) units of packed red blood cells of 300 ml each was administered, in 10 (22%) cases more than 4 units. The median number of units given was 0 (0, 3.5; range 0–16) in placenta accreta and 2.5 (0, 8; range 0–14) in placenta increta and percreta. Blood loss in placenta increta and percreta was not significantly different from that in placenta accreta (*p* = 0.496). When comparing the different types of management, there was no significant difference in blood loss (*p* = 0.061). Within the subgroup placenta increta or percreta, blood loss at prenatal diagnosis was not significantly different to blood loss at intrapartum diagnosis (*p* = 0.327). In addition, with known placenta increta or percreta, there was no significant difference in blood loss with attempted or deferred placental removal (*p* = 0.417). The majority of patients (96%) suffered from anaemia after delivery (haemoglobin < 7 mmol/l), with a median CTCAE grade 2 (1, 2) and a median postpartum haemoglobin value of 5.7 (5.2, 6.3) mmol/l.

**Table 3:** Surgical complications and postpartum morbidity for women with PAS and neonatal outcome

Maternal outcome	Women with PAS (*n* = 46)
Estimated intraoperative blood loss [ml], median (Q1, Q3)	1600 (1100, 2750)
Hysterectomy	18 (39)
Surgical complication (bladder injury), *n* (%)	1 (2)
Anaemia, (CTCAE), *n* (%)	44 (96)
Grade 1 (hgb ≥ 6.2 mmol/l)	12 (26)
Grade 2 (hgb < 6.2–4.9 mmol/l)	24 (52)
Grade 3 (hgb < 4.9 mmol/l)	8 (17)
Endometritis, *n* (%)	1 (2)
Urinary infection, *n* (%)	4 (9)
Postoperative urinary retention with placement or change of ureteral stents, *n* (%)	3 (7)
Central neuroretinal detachment	1 (2)
Abdominal haematoma with revision surgery	1 (2)
Vaginal cuff haematoma with conservative treatment (antibiotics)	1 (2)
Intestinal motility disorder	1 (2)
Temporary sensitive nerve paresis	2 (4)
Persistent proteinuria	1 (2)
Admission to intensive care, *n* (%)	6 (13)
Postpartum length of hospital stay, median (Q1, Q3)	6 (4,8)

Neonatal outcome	Median (Q1, Q3)
Birth weight [g]	2750 ( 2235, 3156)
APGAR score after 5 min	9 (8.25, 10)
Umbilical cord artery pH	7.29 (7.25, 7.35)
Length of stay at intensive care	7 (0,12)
Admission to intensive care, *n* (%)	19 (41)

*PAS* placenta accreta spectrum, *g* grammes, *ml* millilitres, *Q1* 25th percentile, *Q3* 75th percentile, *CTCAE* common terminology criteria for adverse events, *hgb* haemoglobin, *mmol* millimole, *l* litres

The only documented surgical complication, a bladder injury, occurred in a woman who underwent her fifth CS within an emergency delivery due to a prenatal bleeding and suspected foetal distress. Extensive intraabdominal adhesions including the lower anterior uterine wall are described in the corresponding surgical report. One patient with placenta in situ showed clinical signs of endometritis although she got antibiotic treatment. She developed symptoms for sepsis and received a curettage 16 days after delivery and subsequently developed pneumonia with pleural effusions.

There was no case of maternal mortality in the study cohort, and all deliveries were recorded as live births.

## Discussion

In our study, 46 patients were eligible for analysis, resulting in an incidence of 2.49 PAS disorders per 1000 births. The diagnosis was distributed among placenta accreta, increta and percreta with 56, 39 and 4%, respectively. There were a high proportion of women with a history of CS (41%), a present placenta praevia (33%) or an isolated history of curettage (11%). The prenatal detection rate was 33% with 91% of total diagnosis made in our department. The median age of diagnosis was at 35 weeks and delivery at 37 weeks of gestation. The hysterectomy rate was 39%. In 9% of the cases, the placenta was left in situ. 54% of patients required blood transfusion. Blood loss did not differ between cases with versus without prenatal diagnosis (*p* = 0.327). In known cases, an attempt to remove the placenta did not show impact on blood loss (*p* = 0.417).

There is no consensus for a single approach to the treatment of PAS disorders, and optimal management depends on local expertise [25]. Our centre used this scope to offer a variety of options for planned management within a standardised interdisciplinary approach and to adapt them to the individual situation of each patient.

The total incidence of PAS disorders at the study centre was comparable to other published data for tertiary perinatal centres [30], but it was higher than the pooled data of a recent meta-analysis considering studies of the last four decades [2]. One reason for this could be the increasing treatment of PAS disorders in experienced centres [17], which is recommended in the ACOG guidelines [22, 24]. On the other hand, increasing incidences of PAS have been shown for the last decades in the course of rising CS rates [7].

The high proportion of women with a history of CS (41%) as well as the proportion with a present placenta praevia (33%) is remarkable, and these characteristics have been described as major risk factors [7, 31]. In comparison, the proportion of all women delivered at our hospital with a previous CS or present placenta praevia was only 12 and 0.7%, respectively. One in four women with PAS disorders had a history of one or more CSs and a placenta praevia in the current pregnancy. In addition, however, attention should be given to cases with previous curettages. In the study cohort, 11% had neither a placenta previa nor a previous CS but had a history of curettage. In particular, repeated previous curettage should raise awareness of the possibility of a PAS disorder and also encourage targeted screening.

The proportion of placenta accreta of all PAS cases was higher compared to recent cohort studies but lower than in earlier case series [5, 32]. The study centre is a main referral centre in an area with a relatively low CS rate of 24% [33]. The incidence of severe PAS disorders increases with the number of previous CSs [5]. Therefore, the lower proportion of placenta increta and percreta could be due to the lower percentage of performed CSs in the area. Furthermore, in the present analysis, staging was consistently determined according to intraoperative clinical criteria. Here, an underdiagnosis of placenta percreta is possible due to an interpretation as adhesions.

In the study period, one third of all PAS was diagnosed prenatally. Standardised ultrasound criteria are the means of choice for prenatal diagnosis of PAS and can reduce morbidity [34]. However, despite high sensitivity and specificity of ultrasound, placenta accreta is more difficult to detect than placenta increta or percreta as it may not have any specific ultrasound findings [12]. In placenta accreta, there are often no signs of invasion or only placental lacunae, which are often also found in normal placentas. In contrast, placenta increta or percreta is associated with ultrasound signs such as loss of 'clear zone' or bladder wall interruption and evidence of increased vascularity [35, 36]. Thus, studies that exclusively consider the latter show higher detection rates in comparison to all investigated cases [37, 38]. This is also confirmed in our study cohort with 75% prenatally diagnosed placenta increta and percreta. In cases of suspected PAS, new standardised ultrasound markers, as proposed by the European Working Group on Abnormally Invasive Placenta in 2016 [10] in combination with an increased awareness of risk factors have led to a more differentiated sonography approach and documentation in our centre within the study period. However, a higher number of annual cases would be necessary to demonstrate a possible significant improvement in detection rates after the introduction of the new nomenclature.

The median gestational age at delivery was 37 weeks in the entire study cohort, and this represents the upper limit of the international recommendations of the FIGO guidelines [24]. Planned delivery in the absence of risk factors between 36 and 37 weeks of gestation can only be performed in PAS with prenatal diagnosis. In our study cohort, 80% of prepartal diagnoses were made at our centre. Ultrasound signs were detected during ultrasound screening or after referral in the second trimester, especially in the presence of risk factors.

In two thirds (67%) of prenatally diagnosed PAS cases, a planned caesarean hysterectomy was performed (*n* = 10), which is the preferred management option for known PAS according to expert opinion [25]. In the other cases (*n* = 5) of prenatally diagnosed PAS, after delivery of the foetus by CS, the placenta was left in situ. These uterus-preserving ('conservative') and expectant management options for placental delivery during laparotomy have also found entrance into guideline recommendations [22]. Intentional vaginal delivery is not a widely accepted option in known PAS [23].There was no intentional vaginal delivery in cases of prenatal diagnosis in the study cohort either. However, all 16 cases of placenta accreta, each unknown, were successfully managed by vaginal delivery and curettage without laparotomy, as already described elsewhere [39]. Vice versa, there was no case of vaginal delivery with unexpected intrapartum diagnosis of placenta increta or percreta. Extended invasive placentation such as placenta percreta is attributed to deep uterine scars such as caesarean scars [5]. In the literature, an association of more than 2 previous CSs with PAS disorders is described [40]. With a history of more than one previous CS, the study centre helps to usually deliver by CS. Thus, the random finding of placenta increta or percreta after vaginal delivery was hardly to be expected. And this could explain the increased CS rate (65%) in women with PAS disorders compared to the whole patient population at the study site (33%).

Patients with PAS disorders suffered from increased peripartal blood loss and had to be treated with blood transfusions in more than half of the cases (54%) with a median of two units of packed red blood cells. In our cohort, the blood loss recorded was not significantly changed by the type of delivery management. However, less than half of the investigated cases of PAS were placenta increta or percreta. Only in case of placenta increta or percreta, a prenatal detection was likely and a guideline-based planning of delivery management was possible. Also for the subgroup of placenta increta and percreta, a higher morbidity due to blood loss compared to placenta accreta has been demonstrated elsewhere [30]. Therefore, these cases could be more relevant when investigating the effects of the type of treatment on blood loss. But even if only the cases of placenta increta and percreta are considered, there was no significant reduction in blood loss on prenatal detection. The medical care capacity of a tertiary referral centre can certainly partially compensate for unexpected negative clinical courses such as bleeding during PAS. This includes standard operating procedures (e.g. on peripartum haemorrhage) and interdisciplinary treatment by experienced obstetricians, gynaecologists and anaesthetists as well as the availability of transfusion medicine around the clock. However, the small number of prenatally diagnosed cases does not allow any conclusions to be drawn on this subject. Therefore, our data are not suitable to question the value of prenatal diagnosis of PAS for the reduction of maternal morbidity/mortality shown so far [28, 32, 35, 36].

It should be noted that in the present study, the most massive blood losses occurred in situations where no prenatal diagnosis was made and where the attempt to remove the placenta led to a hysterectomy (up to 10,000 ml). Under the controlled conditions of a known diagnosis of placenta increta or percreta, however, attempts to remove the placenta did not lead to a significant increase in blood loss. But intentional placental removal remained the exception in prenatally diagnosed PAS, as recommended in the guideline [24]. The significant rate of placental removal attempts in the total cohort is due to the proportion of vaginal deliveries with curettage in which PAS disorders of lower grade could be treated without laparotomy.

Placenta management did not have a significant effect on the hysterectomy rate in known or unknown PAS cases. In fact, intrapartum primary diagnosis and attempts to remove the placenta resulted in massive bleeding leading to hysterectomy. However, even after vaginal delivery in unknown PAS disorders, organ preservation was achieved by curettage. In all planned caesarean hysterectomies, no attempt of placental removal was made in accordance with the guidelines [24]. However, the majority of the placentas left in situ during CS could be removed by curettage in the puerperium preserving the uterus.

There were several limitations in our study. First, the present analysis has a restricted statistical power due to the number of cases of a still very rare disorder. The diagnosis of PAS disorders was based on intraoperative and clinical findings, whereby the classification as placenta accreta, increta or percreta remained subjective despite orientation to the FIGO standard. However, the derivation of therapeutic decisions from a clinically confirmed diagnosis reflects the relevant situation in practice. Second, due to the retrospective nature of the study, the blood loss reported was an individual estimate rather than a truly measured volume. The preoperative blood loss could be underestimated in curettages and laparotomies, because in vaginal deliveries, it was only measured by weighing cloths and not by suction volume, and detailed measurement of blood loss was performed in case of increased bleeding only. Before starting the measurement, the visually estimated blood loss in the given emergency situation may have been misjudged. In addition, the intraoperative blood loss could be misjudged by mixture of amniotic fluid and loss to the floor. Furthermore, no statement was made about cumulative blood loss in the puerperium, which could lead to further morbidity. Third, a different approach to transfusion, haemostasis and vascular control could influence peripartal blood loss in individual cases.

Fourth, we cannot completely exclude the possibility that prenatally undiagnosed cases were at some point expected as PAS by the treating clinicians and were consecutively treated with earlier consideration of available resources. However, there were no data available to strengthen this hypothesis at all.

Valid evaluations of the outcome of different management strategies in PAS are limited not least by the discrepancy between clinical and pathological diagnosis. Newly developed examination protocols for histological preparations include intraoperative evaluation, guided histologic sampling, immediate postoperative examination and dissection guided by preoperative imaging and could more effectively correlate clinical and pathological assessment [41]. In this respect, classification guidelines have been developed by expert panels, which consider not only the classical diagnosis of PAS in case of hysterectomy or partial myometrial resection specimens but also histological findings in delivered placentas and curettings [42]. The establishment of so-called placenta boards is a promising approach, which allows for a regular retrospective evaluation of sonographically, clinically and pathologically diagnosed cases of PAS in an interdisciplinary team [43].

Efforts have been made to establish uterus-preserving management approaches instead of hysterectomy without increasing complication rates in patients with PAS such as the resective-reconstructive approach as a one-step conservative surgery ([18], [44]). Segmental uterine resection may be one alternative to hysterectomy to preserve fertility and reduce morbidity in case of PAS[45, 46]. The combination of perioperative placental localisation by ultrasound, pelvic devascularisation by placement of intra-arterial balloon catheters, placental non-separation, myometrial excision and uterine repair is known as the Triple-P procedure (Carillo Chandrarahan 2019). It is conceivable that this approach reduces the possibility of recurrence by excising the invasion site.

## Conclusion

In this study, no difference in estimated blood loss was found with different management approaches. Consistent screening in the presence of typical risk factors, thorough delivery planning and a well-coordinated team for surgery are essential to reduce morbidity. The results of prospective studies from experienced centres should be pooled in international databases in order to define standard management strategies that can be adapted to the needs of individual patients.

## Data Availability

The data that support the findings of this study are available from the corresponding author, [AE], upon reasonable request.
